# Effect of attachment type and implant position on the retention of mandibular implant-supported overdentures

**DOI:** 10.34172/joddd.41514

**Published:** 2024-12-14

**Authors:** Alireza Saadati, Farnaz Taghavi-Damghani, Sara Tavakolizadeh, Alireza Hadi

**Affiliations:** ^1^Oral Health Research Center, Health Research Institute, Babol University of Medical Sciences, Babol, Iran; ^2^Department of Prosthodontics, School of Dentistry, Shahid Beheshti University of Medical Sciences, Tehran, Iran

**Keywords:** Attachment, Dental implant, Overdenture, Retention

## Abstract

**Background.:**

In implant-supported overdentures increase in the number of implants improves the retention and stability of the overdentures. A direct correlation exists between prosthesis retention and patient satisfaction. Therefore, this experimental study assessed the effect of attachment type and implant position on the retention of mandibular implant-supported overdentures.

**Methods.:**

A transparent acrylic resin model of the mandible was fabricated, and dental implants were positioned at the first molar area (position 6), between the lateral incisor and canine teeth (positions B and D), and first premolars (positions A and E) bilaterally. Novaloc attachments (strong, medium, and light retentive caps) were used with ABDE, 6AE6, and 6BD6 implant positions. A Dolder bar attachment was also used with the ABDE implant position. Overdenture retention was measured under vertical loading, and the maximum dislodging force (MDF) was recorded. Data were analyzed by two-way and one-way ANOVA and post hoc Tukey tests (α=0.05).

**Results.:**

The effects of attachment type and implant position [except for ABDE and 6AE6 with light retention insert (*P*=0.49), and 6AE6 and 6BD6 with strong retention insert (*P*=0.48)], and their interaction effect were significant on MDF (*P*<0.01). The highest retention was recorded for bar attachment (65.15 N), with the lowest for Novaloc attachment with light retention insert at ABDE implant position (11.97 N).

**Conclusion.:**

With Novaloc attachments, minimum retention was recorded in ABDE, and maximum retention was recorded in the 6BD6 implant position due to the increased distance between attachments. The strong insert showed the highest retention value, which confirmed the manufacturer’s claim. Maximum retention was recorded with the bar and clip attachment.

## Introduction

 The number of edentulous patients is considerably high, even in countries with high oral health standards.^1^ Edentulism has a negative impact on oral and general health and decreases the quality of life.^2,3^ Oral rehabilitation of edentulous patients is currently an essential part of prosthodontic treatments.^4^ An implant-supported overdenture is one of the treatment options to improve patient satisfaction and nutritional status.^5,6^ Various clinical studies have been conducted to assess the effects of implant-supported prostheses on the oral health-related quality of life, demonstrating significant improvements following the insertion of dental implants.^3,7^ It is assumed that increasing the number of implants improves the retention and stability of the overdenture.^8^ A direct correlation exists between prosthesis retention and patient satisfaction.^9^ Implant placement and removable implant-supported overdenture can prevent bone resorption around neighboring bone.^10^ Implant-supported overdentures have three components: dental implants, attachment, and suprastructure.^11^ Selecting the attachment type is important in different cases.^12^ The bar and clip attachment, which is commonly used, and the Novaloc attachment, which has recently been released, are two attachment systems used in this study. The Novaloc retentive system has 6 retention inserts with different retention levels coded with different colors.^13^ The matrix of this attachment can be adapted to two abutments with a maximum of 40º divergence, which is a significant advantage of this system. The matrix can be made of titanium or polyether ether ketone; the latter is used when higher esthetics is required since it has a neutral color.^14^

 It seems that placing anterior implants alone to support the overdenture is not sufficient and causes bone loss in the posterior region; thus, posterior implants may be an option to prevent bone loss.^10^

 With the advances in science, prosthodontic-driven treatments are suggested, which means that implant position is determined based on prosthetic requirements.^15^ Accordingly, implant placement in the posterior mandible is considered.

 Considering all the above, this study aimed to assess the effect of implant position and attachment type on the retention of implant-supported overdentures in the mandible.

## Methods

**Figure 1:**
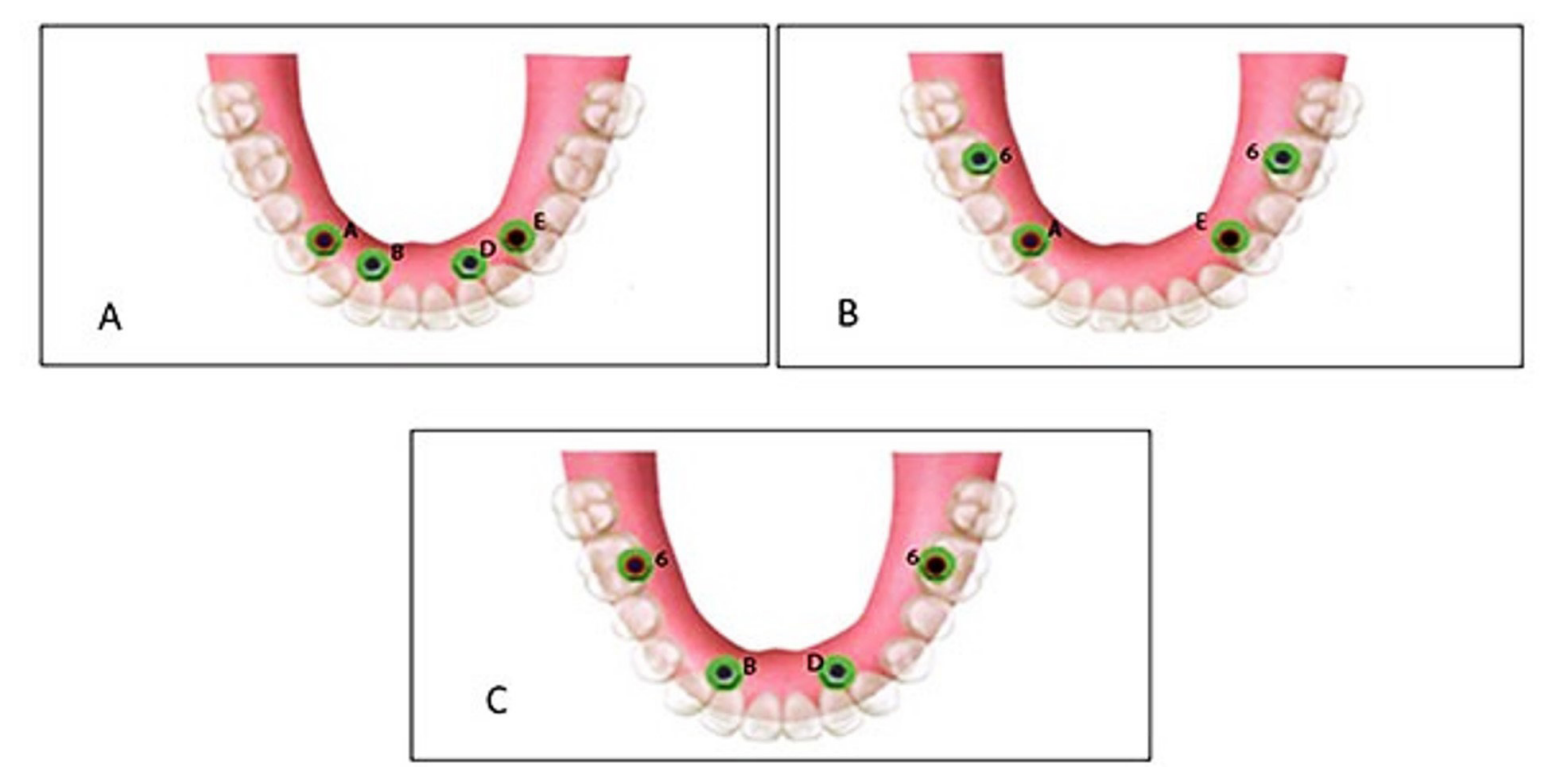
Three positions: ABDE (A), B6AE6 (B), 6BD6 (C)

 The present in vitro study was approved by the Ethics Committee of the Shahid Beheshti University of Medical Sciences, Tehran, Iran (IR.SBMU.DRC.REC.1398.202). It was conducted on a transparent acrylic resin model of an edentulous mandible, in which six tissue-level implants (Standard Plus Implant, Straumann group, Switzerland) measuring 4.1 mm in diameter and 10 mm in length were placed. In each experiment, four implants were activated by the connection of implant and attachment. Three different positions were evaluated as follows (Figure 1):

 ABDE: In this position, four implants were placed bilaterally between the lateral incisor and canine teeth (B and D) and first premolars (A and E).

 6AE6: In this position, four implants were placed bilaterally at the site of the first molars and first premolars (A and E).

 6BD6: In this position, four implants were placed at the site of the first molars and bilaterally between the lateral incisor and canine teeth (B and D).

 Each experiment was repeated five times for each implant position (ABDE, 6AE6, and 6BD6), for each group of light, medium, and strong retention inserts, and bar and clip attachment.

 A control group (the model without attachment) was also considered and underwent testing five times. A total of 60 tests were performed.

 The study was conducted in five steps as follows:

1. Fabrication of model, drilling, and placement of implants
2. Placement of housings in the overdenture
3. Fabrication of load cell
4. Measurements in a universal testing machine
5. Testing

###  Fabrication of model, drilling, and placement of implants

**Figure 2:**
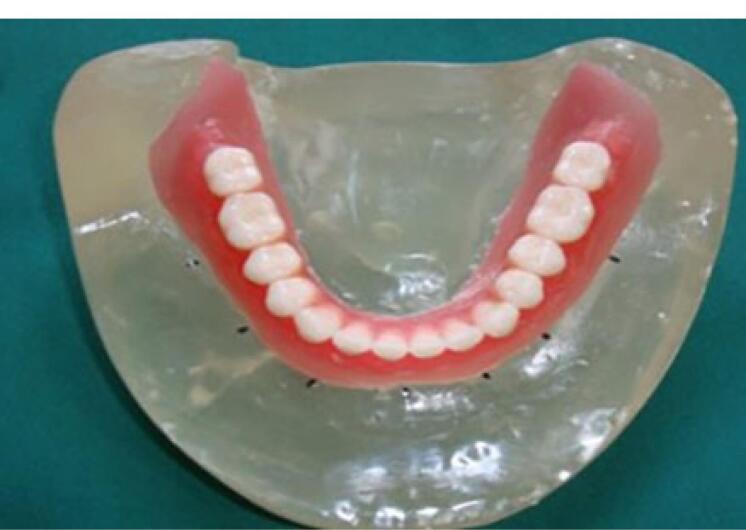
Positions A, B, C, D, E, and right and left first molars were marked on the model

**Figure 3:**
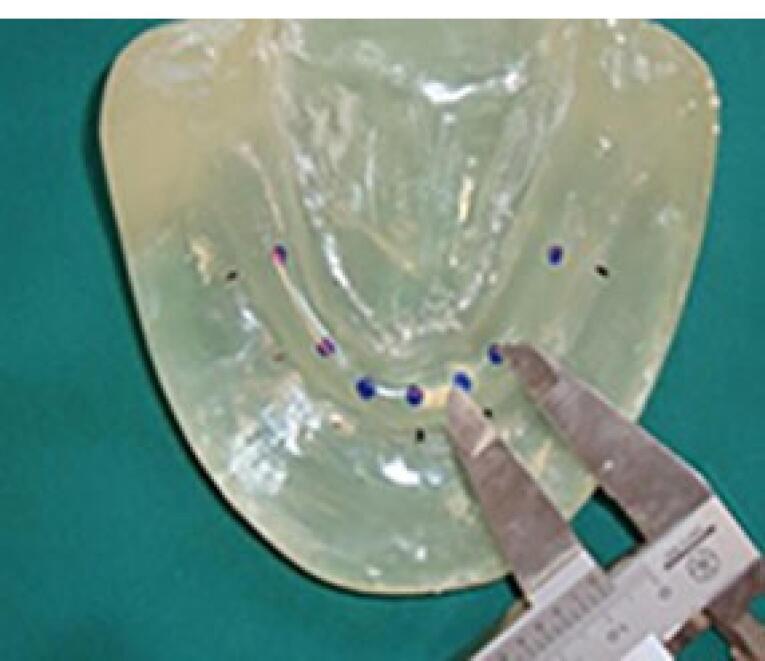
The distance between A-B, B-C, C-D, and D-E was 8 mm, and between 6-A and 6-E was 16 mm

**Figure 4:**
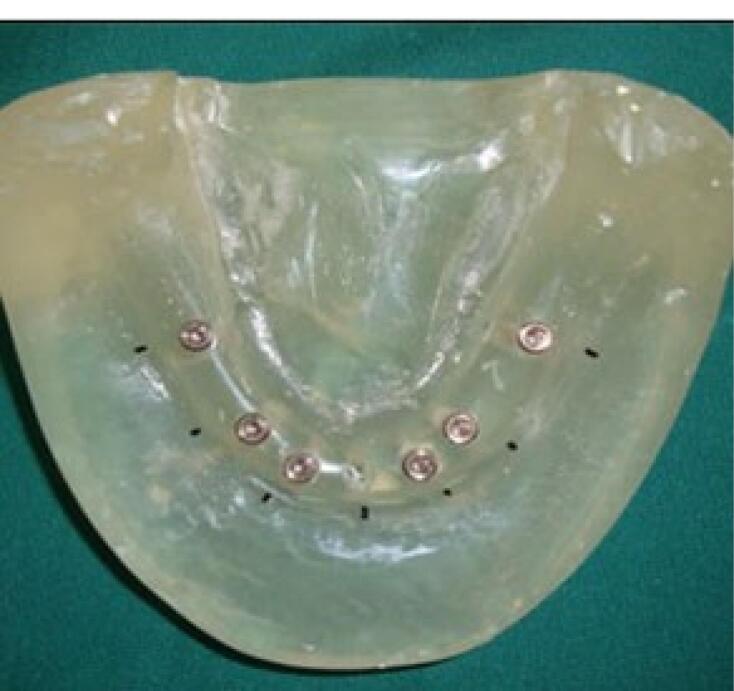
Inserted and fixed implants

**Figure 5:**
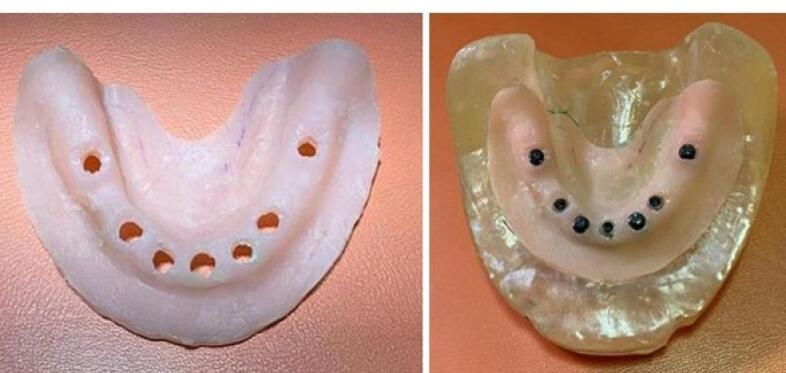
Additional silicone (Gingifast; Zhermak SpA; Germany) was used to simulate the gingiva

 A transparent acrylic resin (Ispringen, Germany) model of the mandible with no undercut was fabricated to better simulate the clinical situation. For this purpose, a mandibular master cast of a 60-year-old male patient with no systemic disease, who had extracted all his teeth about a year ago and had insignificant uniform resorption of the residual ridge, was used. The undercuts were blocked out with wax (Polywax, Turkey) using a surveyor (Ney, USA). The cast borders were extended by 1 to 2 cm all around, and the vestibules were filled with wax. The duplicate cast was then fabricated, and the acrylic resin model was finally fabricated with Doubligel agar (Dandiran, Iran) and Orthocryl auto-polymerizing acrylic resin (Ispringen, Germany). A base plate was manufactured on the cast using visible light-cured (VLC) acrylic resin (Megadenta, Germany), and normal-size artificial teeth (Ideal Maco, Iran) were mounted in a plane parallel to the residual ridge. Accordingly, positions A, B, C, D, E, and right and left first molars were marked (C indicated mandibular symphysis). A and B codes were assigned to locations in the right quadrant, and D and E were assigned to locations in the left quadrant (Figure 2). To ensure the correct position of implant holes based on the location of mounted teeth, the distance between the holes was measured by a caliper (Fowler, Canada) with 0.1 mm accuracy. This distance was 8 mm between A-B, B-C, C-D, and D-E and 16 mm between 6-A and 6-E (Figure 3). Implant holes were drilled by a series of Straumann drills (Straumann Group, Switzerland) using a milling machine (Paraskop M; Bego, Bremen, Germany) to ensure their parallel positions. Six tissue-level implants (Straumann Standard Plus Implant, Straumann group, Switzerland) measuring 4.1 mm in diameter and 10 mm in length were placed by the milling machine. To ensure firm placement of implants, the holes were created by a drill one size smaller than the implant. After the one-time insertion of the implant at the site and ensuring its optimal position, it was removed and fixed again using Super Glue (Razi, Iran) (Figure 4). Additional silicone (Gingifast; Zhermak SpA; Germany) measuring 2 mm in thickness was used to simulate the gingiva (Figure 5). Due to the absence of undercuts, retention could only be obtained from the attachments.

**Figure 6:**
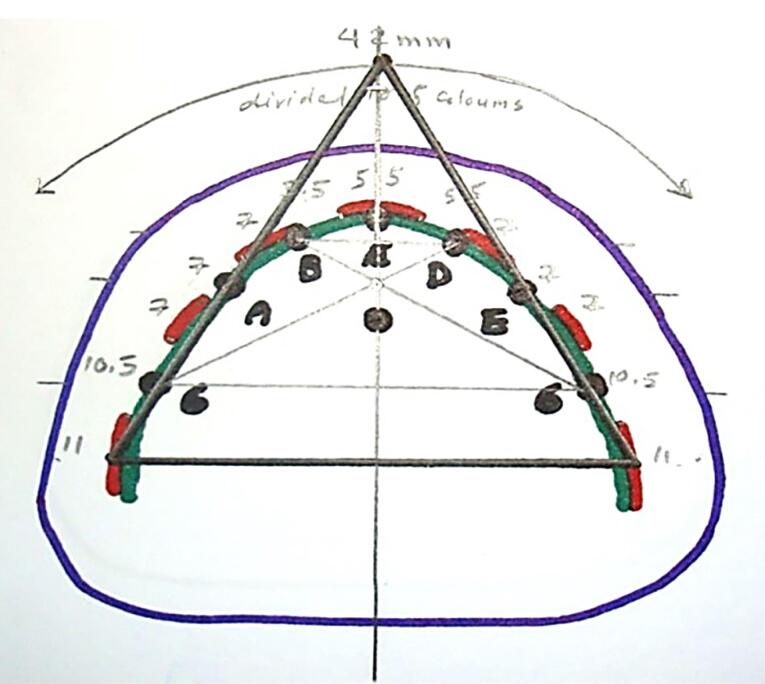
An equilateral triangle was defined to ensure balanced force distribution. The three corners and the center of gravity of this triangle served as the points of load application

**Figure 7:**
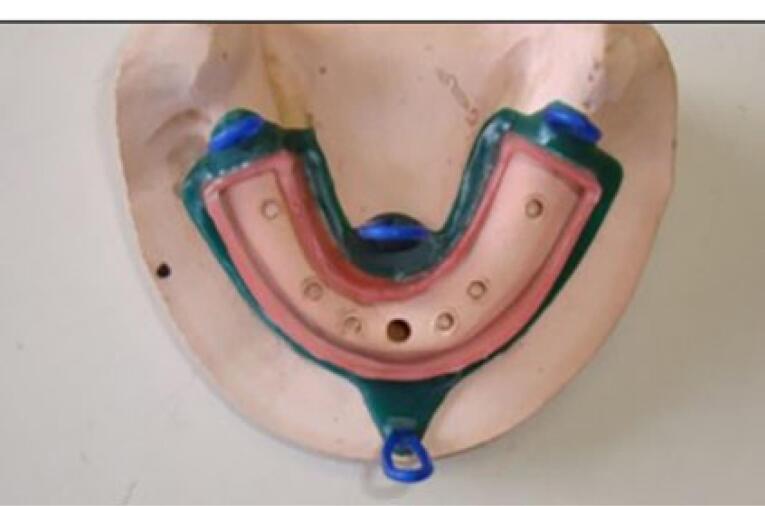
The tunnel comprised of two U-shaped wax components on the buccal and lingual vestibules attached to each other at the end

**Figure 8:**
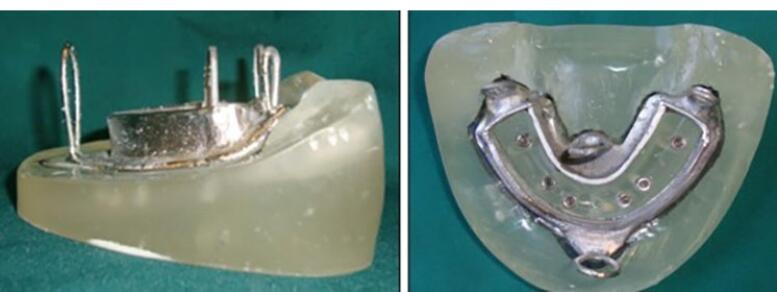
The metal framework

 A chromium-cobalt framework (Biosil F; Dentsply Degudent, Germany) was fabricated.^11,16,17^ To fabricate a metal framework, a duplicate was first manufactured by taking an impression with Doubligel agar (Dandiran, Iran) and pouring it with dental stone (Nanovest Germany). An equilateral triangle measuring 5 cm in length on each side was defined to ensure balanced force distribution. The three corners and the center of gravity of this triangle served as the points of load application (Figure 6). Waxing was performed on this model by designing two U-shaped wax components with 2-mm thickness on the buccal and lingual vestibules attached to each other at the end. This U-shaped assembly is called a “tunnel” in this study (Figure 7). Four hooks were created at four points in the anterior, middle, and right and left lateral sides of the triangle to accommodate the housing. This pattern was cast to metal (Figure 8). Polyester threads (Kiancord, Tehran, Iran) were used to attach the hooks to the load cell and the universal testing machine.

###  Placement of housings in the overdenture 

**Figure 9:**
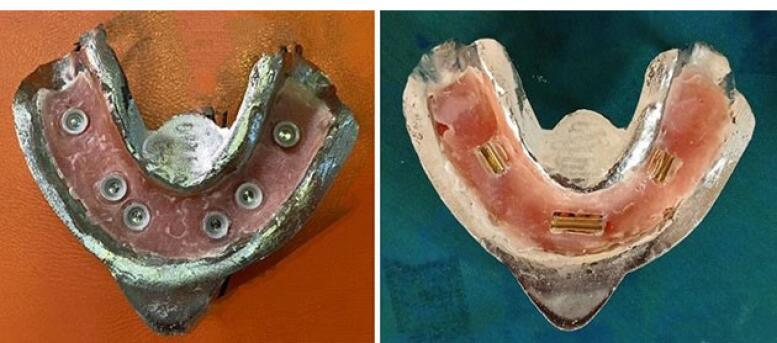
Polished acrylic part

**Figure 10:**
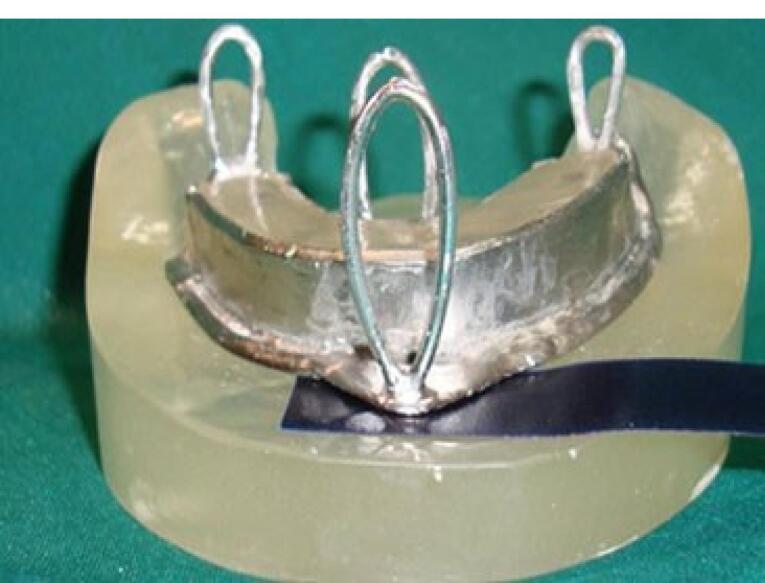
Complete seating of the framework on the model was ensured in the anterior region using 80-µm articulation paper

 After placing the metal framework, the Novaloc abutments (Straumann Group, Switzerland) were tightened (torqued to 20 Ncm), and the housing (Straumann Group, Switzerland) was placed on the abutment. Block-outs were performed around the attachment with wax (Polywax, Turkey). Auto-polymerizing acrylic resin powder and liquid (Meliodent, Iran) were mixed according to the manufacturer’s instructions and applied in the framework on the cast in three parts separately (anterior, right, and left) to minimize acrylic shrinkage. After each step, the framework was placed in a pressure pot (Betadent, Iran). The acrylic part was then polished with dental polishers (Jota, Switzerland). This process was performed separately for two attachment systems (Figure 9). Complete seating of the framework on the model was ensured in the anterior region using 80-µm articulation paper (Coltene/Whaledent, Germany) (Figure 10).

###  Fabrication of load cell 

**Figure 11:**
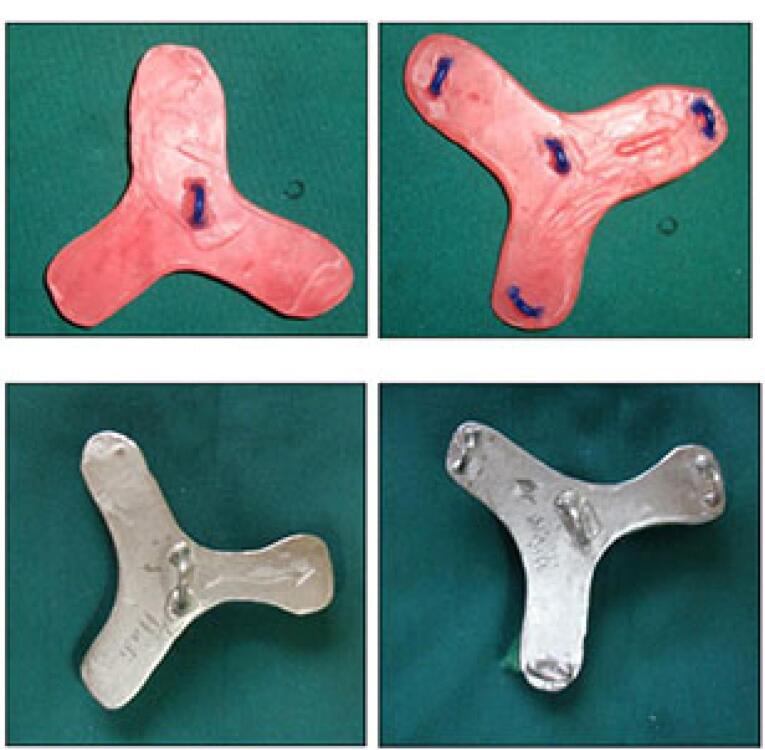
The load cell with four hooks

 To connect the metal framework and its’ attachments to the universal testing machine, a metal plate with three branches was required, known as the load cell. It had four hooks, according to the hooks incorporated in the framework on one side, and another hook right at the center on the other side for connection to a universal testing machine through an S-shaped hook. The load cell was designed and fabricated (Figure 11).

###  Universal testing machine 

**Figure 12:**
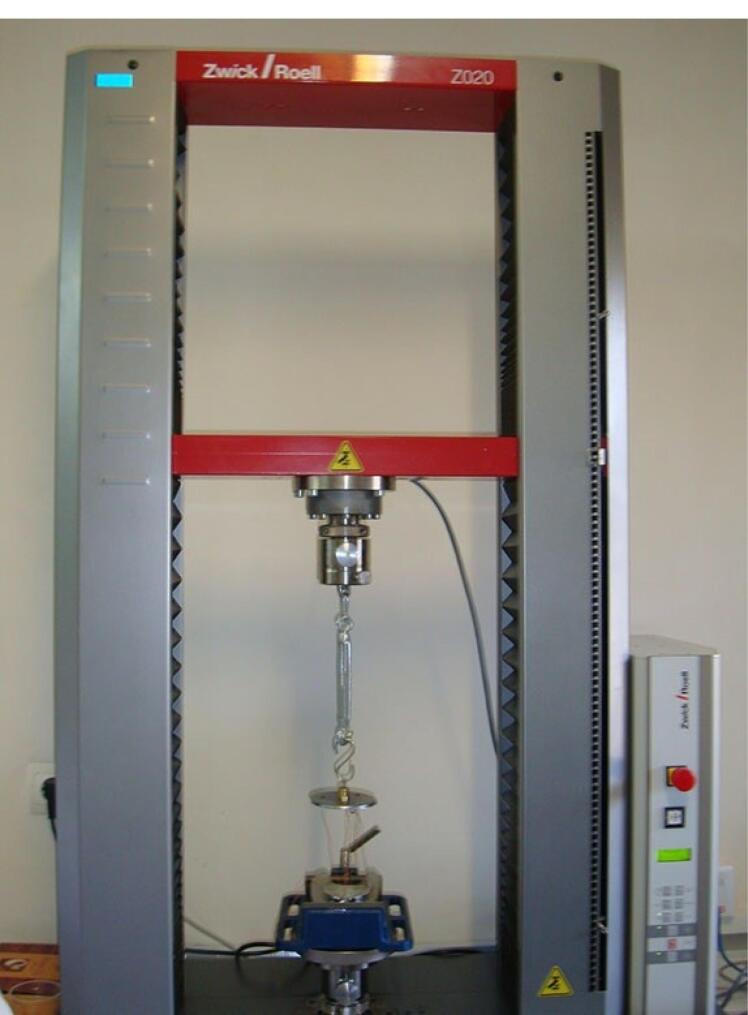
The Universal Testing Machine

 A universal testing machine (Z020; Zwick Roell, Ulm, Germany) was used to measure overdenture retention at different implant positions under vertical loading. The load was applied along the path of insertion of housing and framework at a crosshead speed of 51 mm/minute, according to the speed of denture movement in mastication.^11,17^ The model was fixed to the machine with a clamp. An S-shaped hook measuring 15.5 mm in length was connected to the center of the load cell. On the other side, a polyester thread measuring 0.407 mm in diameter passed through each hook (Figure 12). The maximum dislodging force (MDF) was recorded in Newtons (N) as the force causing complete separation of overdenture from the attachment.^11^

###  Testing

 Each of the three implant positions (ABDE, 6BD6, and 6AE6) was tested with light, medium, and strong Novaloc attachment system retention inserts. Also, the bar and clip attachment (regular Dolder bar and regular Dolder bar matrix) with ABDE implant position, and a control group were tested. Each position was tested five times (a total of 60 tests).^11,16,17^

###  Statistical analysis

 Normal distribution of data was ensured by the Shapiro-Wilk test. The equality of variances was analyzed using Levene’s test. Two-way ANOVA was applied to assess the effect of implant position and attachment type on MDF. The Tukey test was used for pairwise comparisons. One-way ANOVA was applied to analyze the interaction effect of attachment type, retention insert, and implant position on MDF. *P* < 0.05 was considered statistically significant.

## Results

Table 1 presents the measures of the MDF in different implant positions with different retention inserts. As indicated, the highest mean MDF was recorded for the 6BD6 position and strong retention insert (49.13 ± 3.11 N). The lowest mean MDF was recorded for the ABDE position with light retention insert (11.97 ± 0.78 N).

**Table 1:** Measures of the MDF in different implant positions with different retention inserts

**Implant position**	**Retention Insert**	**Number**	**Mean**	**SD**	**Minimum**	**Maximum**
6AE6	Light	5	14.3600	1.84357	11.62	16.66
	Medium	5	34.7220	1.76651	32.19	37.07
	Strong	5	46.7300	2.41426	44.02	49.91
	Total	15	31.9373	13.95680	11.62	49.91
6BD6	Light	5	21.1640	2.12675	18.69	24.13
	Medium	5	36.3520	3.60492	30.78	40.15
	Strong	5	49.1340	3.11087	44.53	52.54
	Total	15	35.5500	12.15792	18.69	52.54
ABDE	Light	5	11.9780	0.78954	11.04	12.83
	Medium	5	22.0700	1.77178	19.14	23.81
	Strong	5	34.1500	2.97248	30.40	38.29
	Total	15	22.7327	9.57183	11.04	38.29
Total	Light	15	15.8340	4.32131	11.04	24.13
	Medium	15	31.0480	7.01120	19.14	40.15
	Strong	15	43.3380	7.29462	40.40	52.54
	Total	45	30.0733	12.95928	11.04	52.54
Bar		5	65.1520	4.31814	57.72	68.57

 The difference in the mean MDF was significant among the three implant positions (*P <*0.05), such that maximum force was recorded in the 6BD6 position (35.55 ± 12.15 N), and minimum force was recorded in the ABDE position (22.73 ± 9.57 N). The difference in the mean MDF was also significant among the three retention inserts (*P* < 0.05) such that the maximum force was noted for the strong retention insert (43.33 ± 7.29 N) and minimum force was noted for the light retention insert (15.83 ± 4.32 N).

 Two-way ANOVA showed that the interaction effect of implant position (ABDE, 6AE6, and 6BD6) and type of retention insert (strong, medium, and light) was significant on MDF (*P* < 0.05).

Table 2 presents pairwise comparisons of retention inserts in different implant positions regarding MDF, and Table 3 shows pairwise comparisons of implant positions based on the type of retention insert.

**Table 2:** Pairwise comparisons of retention inserts in different implant positions regarding MDF

**Implant position**	**Retention Insert**		**Mean difference**	***P*** ** value**
	**1**	**2**		
6AE6	Light	Medium	-20.362	< 0.01
		Strong	-32.370	< 0.01
	Medium	Light	20.362	< 0.01
		Strong	-12.008	< 0.01
	Strong	Light	32.370	< 0.01
		Medium	12.008	< 0.01
6BD6	Light	Medium	-15.188	< 0.01
		Strong	-27.970	< 0.01
	Medium	Light	15.188	< 0.01
		Strong	-12.782	< 0.01
	Strong	Light	27.970	< 0.01
		Medium	12.872	< 0.01
ABDE	Light	Medium	-10.092	< 0.01
		Strong	-22.172	< 0.01
	Medium	Light	10.092	< 0.01
		Strong	-12.080	< 0.01
	Strong	Light	22.172	< 0.01
		Medium	12.872	< 0.01

**Table 3:** Pairwise comparisons of implant positions based on the type of retention insert

**Retention insert**	**Position**		**Mean difference (2-1)**	***P*** ** value**
	1	2		
Light	6AE6	6BD6	-6.804	< 0.01
		ABDE	2.382	0.494
	6BD6	6AE6	6.804	< 0.01
		ABDE	9.186	< 0.01
	ABDE	6AE6	-2.382	0.494
		6BD6	-9.186	< 0.01
Medium	6AE6	6BD6	-1.630	< 0.01
		ABDE	12.652	< 0.01
	6BD6	6AE6	1.630	< 0.01
		ABDE	14.282	< 0.01
	ABDE	6AE6	-12.652	< 0.01
		6BD6	-14.282	< 0.01
Strong	6AE6	6BD6	-2.404	0.483
		ABDE	12.580	< 0.01
	6BD6	6AE6	2.404	0.483
		ABDE	14.984	*P* < 0.01
	ABDE	6AE6	-12.580	*P* < 0.01
		6BD6	-14.984	*P* < 0.01

**Figure 13:**
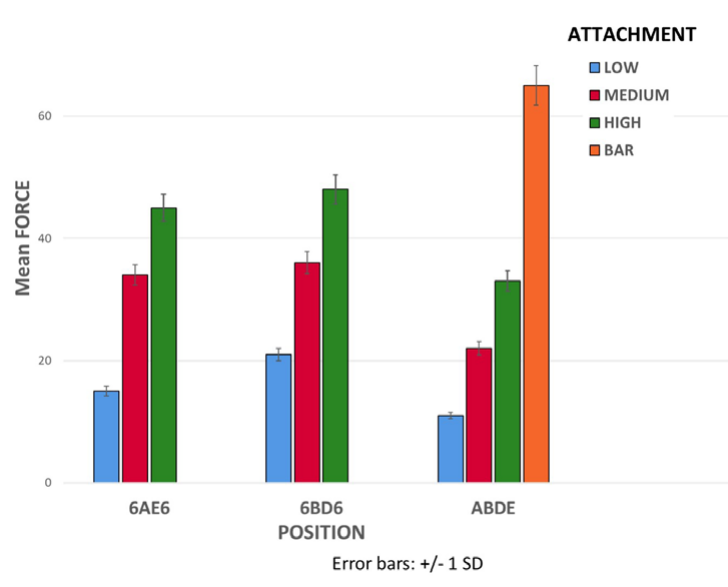
Comparison of MDF in the use of bar and clip attachment in ABDE implant position with Novaloc retention inserts with three different implant positions

 Comparison of MDF in the use of bar and clip attachment in ABDE implant position with Novaloc retention inserts with three different implant positions (Figure 13) showed that the MDF in the use of bar and clip attachment (65.15 ± 4.31 N) was significantly higher than all other groups (*P* < 0.01 for all).

## Discussion

 This study assessed the effect of implant position and attachment type on the retention of implant-supported overdentures in the mandible. The results showed significant effects of implant position and attachment type on retention. The highest MDF was found in the bar and clip attachment group (65.15 ± 4.31 N), which was significantly higher than the value in all the other groups, consistent with the results of Savabi et al^18^ and Tabatabaian et al^17^ regarding the significant effect of attachment type on the retention and stability of prostheses, and in contrast to the findings of Anas El-Wegoud et al,^19^ who found no significant difference between ball and bar attachment systems regarding retention of prostheses. Also, Sabouri et al^20^ reported higher retention of stud (ball) attachments compared with bar attachment, which was different from the present findings. The discrepancy in the results can be due to using a different type of stud attachment in their study. Also, they only evaluated the retention of stud attachments in the ABDE implant position. However, similar to the present study, Gonçalves et al^21^ showed higher retention of bar attachments compared with stud (ball) attachments.

 The highest difference in MDF between bar attachment and Novaloc attachments was noted in ABDE implant position, which was 53.17 N for light, 43.08 N for medium, and 31.00 N for strong retention inserts. The lowest difference in MDF between the bar attachment and Novaloc attachments was noted in the 6BD6 implant position, which was 43.98 N for light, 28.80 N for medium, and 16.01 N for strong retention inserts. In Novaloc attachments, maximum retention was recorded in the 6BD6 implant position, while minimum retention was noted in the ABDE implant position. Similar results were reported by Scherer et al^22^ regarding the effect of the number, distribution, and position of implants on the retention and stability of mandibular overdentures. Consistent with the present results, Shaarawy and Aboelross^23^ demonstrated that more posterior placement of implants relative to the inter-foraminal region was more suitable for loading of mandibular implant-supported overdentures due to the reduction in electromyography activity of masseter and temporalis muscles.

 Higher MFD in 6AE6 and 6BD6 implant positions, compared with ABDE, indicated the effect of the first molar position on the retention and stability of implant-supported overdentures. Similarly, Sadr et al^24^ indicated that implant placement in 6AE6 and 6BD6 positions provided higher retention for the prosthesis. In the comparison of 6AE6 and 6BD6 positions, 6BD6 showed higher retention. Sadr et al^24^ demonstrated that more distal placement of implants increased retention, which was consistent with the present results and can be attributed to the farther distribution of implants relative to each other.

 In the present study, the difference in MDF was significant in the three types of retention inserts in all implant positions, consistent with the manufacturer’s claim regarding the presence of a significant difference in retention between different types of retention inserts. However, the calculated ratios for MDF of medium and strong retention inserts to light retention inserts were 2.41 and 3.25 in the 6AE6 implant position, 1.71 and 2.32 in the 6BD6 implant position, and 1.84 and 2.85 in the ABDE implant position, respectively. These ratios were different from those reported by the manufacturer (1.6 for medium compared with light, and 2.2 for strong compared with light), which may be attributed to the manufacturer’s different method of conduction of the test and the interaction effect of implant position and retention insert type.

 We found no study that evaluated the effect of implant position on retention of Novaloc attachments. In light retention inserts, the difference in retention was not significant between 6AE6 and ABDE implant positions. However, this difference was significant for light retention inserts in other implant positions, and maximum retention was noted in the 6BD6 implant position. In medium retention inserts, the difference in MDF was significant in all implant positions, and the highest MDF was recorded in the 6BD6 position. In contrast, the lowest retention was recorded in the ABDE position. Higher MDF in the 6BD6 position can be attributed to the increased distance between attachments. In strong retention inserts, the difference in MDF was not significant between 6AE6 and 6BD6 positions. This difference was significant in other positions, and the highest MDF was recorded in the 6BD6 position. Insignificant differences between implant positions in light, medium, and strong retention inserts can be attributed to the significant interaction effect of implant position and retention insert.

 This study had an in vitro design. Thus, the results cannot be well generalized to the clinical setting. Future studies with different types of bars and implant positions are recommended. Also, the effects of bar height and abutment height (different gingival heights) must be investigated in further studies. The anteroposterior and lateral stability of overdentures should be assessed as well. Furthermore, other attachment systems should be evaluated, and finite element analysis is recommended for a more accurate evaluation of stress distribution patterns in bone.

## Conclusion

 Within the limitations of this study, it might be concluded that bar and clip attachment is recommended when the aim is to achieve maximum retention for the overdenture. When the application of bar attachment is not clinically possible, implants are recommended to be placed at the 6BD6 position to achieve maximum retention. Increased inter-implant distance in the use of stud attachments increases retention. Also, implant placement at the first molar region increases the overdenture retention compared with other positions.

## Competing Interests

 The authors declare no conflicts of interest.

## Ethical Approval

 This study was approved by the Ethics Committee of Shahid Beheshti University of Medical Sciences with an ethical number of IR.SBMU.DRC.REC.1398.202. There is no conflict with ethical considerations.
